# Ghrelin Receptor (GHS-R1A) Antagonism Suppresses Both Alcohol Consumption and the Alcohol Deprivation Effect in Rats following Long-Term Voluntary Alcohol Consumption

**DOI:** 10.1371/journal.pone.0071284

**Published:** 2013-08-20

**Authors:** Petra Suchankova, Pia Steensland, Ida Fredriksson, Jörgen A. Engel, Elisabet Jerlhag

**Affiliations:** 1 Institute of Neuroscience and Physiology, Department of Pharmacology, The Sahlgrenska Academy at the University of Gothenburg, Gothenburg, Sweden; 2 Department of Clinical Neuroscience, Division of Psychiatry, Karolinska Institutet, Stockholm, Sweden; University of South Florida, United States of America

## Abstract

Alcohol dependence is a heterogeneous disorder where several signalling systems play important roles. Recent studies implicate that the gut-brain hormone ghrelin, an orexigenic peptide, is a potential mediator of alcohol related behaviours. Ghrelin increases whereas a ghrelin receptor (GHS-R1A) antagonist decreases alcohol consumption as well as operant self-administration of alcohol in rodents that have consumed alcohol for twelve weeks. In the present study we aimed at investigating the effect of acute and repeated treatment with the GHS-R1A antagonist JMV2959 on alcohol intake in a group of rats following voluntarily alcohol consumption for two, five and eight months. After approximately ten months of voluntary alcohol consumption the expression of the GHS-R1A gene (*Ghsr*) as well as the degree of methylation of a CpG island found in *Ghsr* was examined in reward related brain areas. In a separate group of rats, we examined the effect of the JMV2959 on alcohol relapse using the alcohol deprivation paradigm. Acute JMV2959 treatment was found to decrease alcohol intake and the effect was more pronounced after five, compared to two months of alcohol exposure. In addition, repeated JMV2959 treatment decreased alcohol intake without inducing tolerance or rebound increase in alcohol intake after the treatment. The GHS-R1A antagonist prevented the alcohol deprivation effect in rats. There was a significant down-regulation of the *Ghsr* expression in the ventral tegmental area (VTA) in high- compared to low-alcohol consuming rats after approximately ten months of voluntary alcohol consumption. Further analysis revealed a negative correlation between *Ghsr* expression in the VTA and alcohol intake. No differences in methylation degree were found between high- compared to low-alcohol consuming rats. These findings support previous studies showing that the ghrelin signalling system may constitute a potential target for development of novel treatment strategies for alcohol dependence.

## Introduction

Alcohol dependence, a chronic, relapsing brain disorder, is one of our societies major public health problems [1] and the clinical efficacy of the available pharmaceutical agents is limited [2]. Development of alcohol dependence largely depends on the effects of alcohol on the brain reward system, i.e. the mesocorticolimbic dopamine system including the ventral tegmental area (VTA), nucleus accumbens (N.Acc.), prefrontal cortex (PFC), hippocampus and amygdala (for review see [3]–[6]). However, by elucidating the indirect neurochemical mechanisms through which alcohol activates the brain reward system, novel treatment strategies can be developed. Recently ghrelin, a circulating gut-brain hormone, was introduced as a potential target for the treatment of alcohol dependence [7]–[10].

Ghrelin increases food intake [11], [12] as well as appetite [13] via hypothalamic growth hormone secretagogue receptors (GHS-R1A)) (for review see [14]). However, several studies show that central GHS-R1A are expressed outside of the hypothalamus implying that ghrelin has important physiological functions besides body weight regulation. Indeed, GHS-R1A are expressed throughout the mesocorticolimbic dopamine system [15], [16] and it has recently been suggested that ghrelin may have a role in reinforcement as well as reward-seeking behaviour [7], [10], [17]. This hypothesis is supported by preclinical studies showing that ghrelin activates the mesocorticolimbic dopamine system [18]–[22] and that central ghrelin signalling is involved in the regulation of the rewarding properties of addictive drugs including alcohol [7], [23]–[29]. In addition, ghrelin increases, whereas GHS-R1A antagonists decrease alcohol consumption as well as operant self-administration of alcohol in rodents that have consumed alcohol for twelve weeks [7], [9], [10]. Interestingly, emerging data show that the roles of several gut-brain hormones extend beyond food intake regulation to include the control of alcohol intake. For instance, glucagon-like peptide 1, leptin and galanin all reduce food, as well as alcohol consumption in rodents [30]–[33].

In addition to GHS-R1A, an important role of nicotinic acetylcholine receptors for the reinforcing properties of ghrelin, as well as alcohol, has been suggested. Indeed ghrelin and alcohol induced reward appear to be mediated via the α3β2 rather than α4β2 subtypes of the nicotinic acetylcholine receptors in the VTA [34]–[36]. Supportively, pharmacological suppression of the α3β2 subtypes reduced alcohol intake in rodents [35], [37].

To further elucidate the role of ghrelin signalling in alcohol mediated behaviours, the present study was designed to investigate the effect on alcohol intake following treatment with the GHS-R1A antagonist JMV2959, in rats that voluntarily had been drinking alcohol for two, five and eight months. In a separate group of rats we evaluated the effect of a JMV2959 on relapse, an important part of alcohol dependence, using the alcohol deprivation paradigm in rats [38]. To evaluate the effects of long-term voluntary alcohol intake on the central ghrelin system, we examined the expression of the GHS-R1A gene, *Ghsr*, as well as α3, α4 and β2 nicotinic acetylcholine receptor subtype genes in reward related areas including the VTA, N.Acc., PFC, hippocampus and amygdala in rats after approximately ten months of voluntary alcohol consumption.

## Materials and Methods

### Animals

Nineteen adult male Rcc Han Wistar rats (Harlan, Netherlands), were used for the acute and repeated JMV2959 treatment study and thirty male adult Wistar rats (Taconic Farms A/S, Ejby, Denmark) were used for the alcohol deprivation effect study. All animals were allowed to habituate to the animal facilities before initiation of the experiment. They were individually housed in Plexiglas cages and were maintained at 20°C with 50% humidity. All rats were maintained on a 12 hour reversed light dark cycle (lights off at 10am). Tap water and food (Normal chow; Harlan Teklad, Norfolk, England) were supplied *ad libitum*, for all rats. The study was carried out in strict accordance with the recommendations in the Swedish Animal Welfare Act and was approved by the Swedish Ethical Committee on Animal Research in Stockholm, Sweden (Dnr: N381/08) and the Ethics Committee for Animal Experiments in Gothenburg, Sweden (permit numbers: 80–07, 19–09). All efforts were made to minimize suffering.

### Drugs

The 96% alcohol (Solveco AB, Stockholm, Sweden) was diluted to a 20% vol/vol solution using tap water for intermittent access 20% alcohol two-bottle-choice drinking paradigm. JMV2959 (generously provided by Æterna Zentaris GbmH, Frankfurt am Main, Germany) has in previous studies been established as a full GHS-R1A antagonist [39] with no affinity on the dopamine receptors (D1, D2L and D2S receptors) [24]. JMV2959, when administered peripherally, suppresses food intake induced by ghrelin or by the GHS-R1A agonist, hexarelin [39], [40]. The selected doses of JMV2959 (1 and 3 mg/kg) has previously been used in our studies (intraperitoneally, i.p.) [10], [41] and did not affect the gross behaviour of the rats in the previous nor in the present experiments. In all experiments JMV2959 was dissolved in vehicle (0.9% sodium chloride) and administered i.p. (5 ml/kg body weight).

### Intermittent Access 20% Alcohol Two-Bottle-Choice Drinking Paradigm

The intermittent access 20% alcohol two-bottle-choice drinking paradigm induces voluntary intake of high amounts of alcohol [42], [43] and pharmacological relevant blood alcohol concentrations [42], [44]. In brief, rats (n = 19) were given access to one bottle of 20% alcohol and one bottle of water for three 24-hour-sessions per week (Mondays, Wednesdays and Fridays), approximately 10 minutes after the lights went out in a reversed light/dark cycle room. The rats had unlimited access to two bottles of water between the alcohol-access-periods. Bottles were weighed at 1, 4 and 24 hours after the fluids were presented and measurements were taken to the nearest 0.1 gram. The body weight of each rat was measured daily prior to bottle presentation, for calculating the grams of alcohol intake per kilogram of body weight (g/kg). The preference for alcohol over water (the ratio of alcohol to total fluid intake) was calculated at all time points.

### Effects of acute and repeated JMV2959 on voluntary alcohol consumption in rats

The effects of acute JMV2959 administration were evaluated when rats (n = 19) had voluntarily consumed 20% alcohol for two and five months, respectively, using the intermittent-access-two-bottle-choice paradigm [42]. Within each test occasion, all animals received all treatments (JMV2959 1 or 3 mg/kg, or vehicle, i.p.) according to a within-subject, Latin square design and each injection was given 7 days apart. All injections were given 20 minutes before the rats were given access to alcohol and water.

When the rats in the acute JMV2959 evaluation had voluntarily consumed alcohol for a total of approximately eight months, they were subjected to repeated JMV2959-treatment. The rats were divided into two groups with equal baseline alcohol consumption. Over a 10-day-period, the two groups received eight JMV2959 (3 mg/kg) or vehicle injections, respectively (daily injections Mon-Fri and Mon-Wed the succeeding week). All injections were given 20 minutes before the rats were given access to alcohol and water. After the last injection, all rats were subjected to a three-week-wash-out-period with regular intermittent access to 20% alcohol as described above. Thereafter, the administration schedule was repeated with treatments reversed between the groups. Thus, a within-subject design was used. Approximately four weeks after the last injection (approximately ten months of voluntary alcohol consumption in total), all 19 rats were decapitated and the brains were collected for gene expression and DNA methylation analysis as described below.

### Effects of acute GHS-R1A antagonist treatment on the alcohol deprivation effect in rats

The alcohol deprivation model is based on the observation that voluntary alcohol intake will increase temporarily when compared to baseline drinking conditions following forced abstinence in alcohol-experienced rats [38]. Rats (n = 30 in total) were subjected to the intermittent access 20% alcohol two-bottle-choice drinking paradigm (as described above) for seven weeks and a stable baseline alcohol intake (g/kg/day) was obtained. Alcohol-, water-, and food intake was measured at one and 24 hours during baseline as well as following drug treatment. The rats were deprived of alcohol for ten days and alcohol was thereafter reintroduced. Twenty minutes before the reintroduction of alcohol, the rats were treated with either JMV2959 (3 mg/kg, i.p.; n = 15) or vehicle (n = 15) in a balanced design. Thereafter the intermittent access paradigm was resumed for three days and the daily 24 hour measurement of alcohol, food and water intake was taken. The rats were untreated these last three days.

### RNA and DNA isolation

The brains from all rats (n = 19) subjected to the acute, and subsequently, repeated JMV2959 treatment were collected after approximately ten months of voluntary alcohol consumption. The VTA, N.Acc., PFC, hippocampus and amygdala were rapidly dissected and immediately put on dry ice and then stored at −80°C until further processing. Frozen tissue samples were placed in an eppendorf tube and disrupted using a bead mill (TissueLyser, Qiagen, Hilden, Germany) with stainless steel beads (5 mm, Qiagen). Total RNA was extracted in an automated sample preparation robot (QIAcube, Qiagen) using the AllPrep DNA/RNA Mini Kit (Qiagen). The quality and concentration of the RNA and DNA samples were assessed using a NanoDrop (Thermal Scientific, Odessa, TX, USA). Due to low A260/230 ratios for the majority of samples they were all purified using the QIAamp DNA Mini Kit (Qiagen) and then reassessed using the NanoDrop.

### Gene expression analysis

The preparation of cDNA was done using the QuantiTect Reverse Trascription Kit (Qiagen). TaqMan predesigned primers and probes were ordered from Applied Biosystems (Foster City, CA, USA). The target genes were *Ghsr* (assay number: Rn00821417_m1), nicotinic acetylcholine receptor subtype α3, α4 and β2 (assay numbers: Rn00583820_m1, Rn00577436_m1 and Rn00570733_m1, respectively) and two endogenous controls were selected (*Gapdh*: Rn01775763_g1, *Actb*: Rn00667869_m1). TaqMan Gene Expression Master Mix (Applied Biosystems) was used in a quantitative real time polymerase chain reaction (qRT-PCR) performed on an ABI 7900HT (Applied Biosystems). The data obtained was analysed using the comparative C_T_ method, described in detail previously [45], where the group of low alcohol consuming rats was set as the calibrator. ΔC_T_ values where obtained by subtracting the threshold cycle (C_T_) of the endogenous control gene from that of the target gene. ΔΔC_T_ where then calculated by subtracting the mean ΔC_T_ of the calibrator from the ΔC_T_ of the target gene for each subject. Relative quantities to the calibrator were calculated as Fold  = 2^−ΔΔC^_T_.

### Methylation analysis

Because a negative correlation between *Ghsr* expression and alcohol intake was found only in the VTA, this area was also analysed in the DNA methylation experiment. The DNA samples extracted from the VTA were bisulfite converted using the EpiTect Fast DNA Bisulfite Kit (Qiagen). The PyroMark PCR kit was used with the pre-designed assays covering a CpG island in the *Ghsr* gene (PM00525532; Qiagen), consisting of four CpG sites. Optimisation of the PCR resulted in an annealing temperature of 56°C. For the pyrosequencing step, PyroMark Gold reagents (Qiagen) were used and the analysis was performed on a pyrosequencer PSQ96 MA (Qiagen) with the PyroMark Q96 Software 2.5.

### Statistical analysis

A three period balanced cross-over design was used to evaluate the effect of acute JMV2959 treatment on alcohol and water consumption, and alcohol preference. The effects of treatment and test occasion (two and five months of voluntary alcohol consumption before treatment, respectively) were analysed using multifactorial ANOVA for repeated measures. Treatment and Test Occasion were entered in the ANOVA as within-subject factors. It was planned before the experiment to compare the treatment differences within each time point at each test occasion if there was an overall main effect on treatment (*P*<0.05). If the interaction of Treatment*Test Occasion displayed a *P*-value less than 0.05, appropriate planned comparisons were carried out between test occasions.

In the repeated treatment two-bottle-choice experiments, the first three drinking sessions post each treatment period were included in the statistical analysis to evaluate the effects on rebound drinking. Treatment and day were entered into the ANOVA as within-subject factors. Prior to the experiment it was planned to carry out tests between treatment differences within each time point and day if the main effect of treatment and the interaction day*treatment showed *P*<0.05. Greenhouse-Geisser adjustments were used when sphericity assumption was violated.

The baseline drinking in rats later exposed to an alcohol deprivation period was evaluated by one-way ANOVA. The effect of treatment on the alcohol deprivation effect was analysed with a two-way ANOVA. To compare the effects of treatment on intake at each time point an independent sample t-test was used.

An independent sample t-test was applied to the 2^−ΔΔC^_T_ values to explore the impact of long-term alcohol consumption on *Ghsr* gene expression in rats. The estimated effects of alcohol consumption from the independent sample t-test were expressed as up or down folds. A fold change reduction (i.e. down-fold and a fold change <1) was calculated by taking the negative inverse of 2^−ΔΔC^_T_. Methylation analyses were run for those genes and brain regions for which significant results in the expression analysis were recorded. Mean overall methylation percentage for the CpG sites included in the analysis was calculated. Methylation differences between high- and low-alcohol consuming rats were assessed using independent sample T-test. In addition, overall methylation of the gene was correlated against gene expression data using Pearson product-moment correlation coefficient. Correction for multiple testing was carried out using the Bonferroni principle in which the *P*-values from the expression and methylation analysis were multiplied by number of investigated target genes (i.e. five) and CpG sites (i.e. four), respectively. Corrected *P*-values are designated *P_c_*.

The consumption data are presented as mean ± SEM, whereas the expression and methylation data are represented as mean ± SD. A probability value of *P*<0.05 was considered as statistically significant. IBM SPSS statistics (version 20.0, SPSS Inc., Chicago, Illinois) or GraphPad Prism 5.0 (GraphPad Software, La Jolla, CA, USA) was used for the analyses.

## Results

### Acute administration of the GHS-R1A antagonist JMV2959 selectively decreases voluntary alcohol consumption in rat

**Figure 1:**
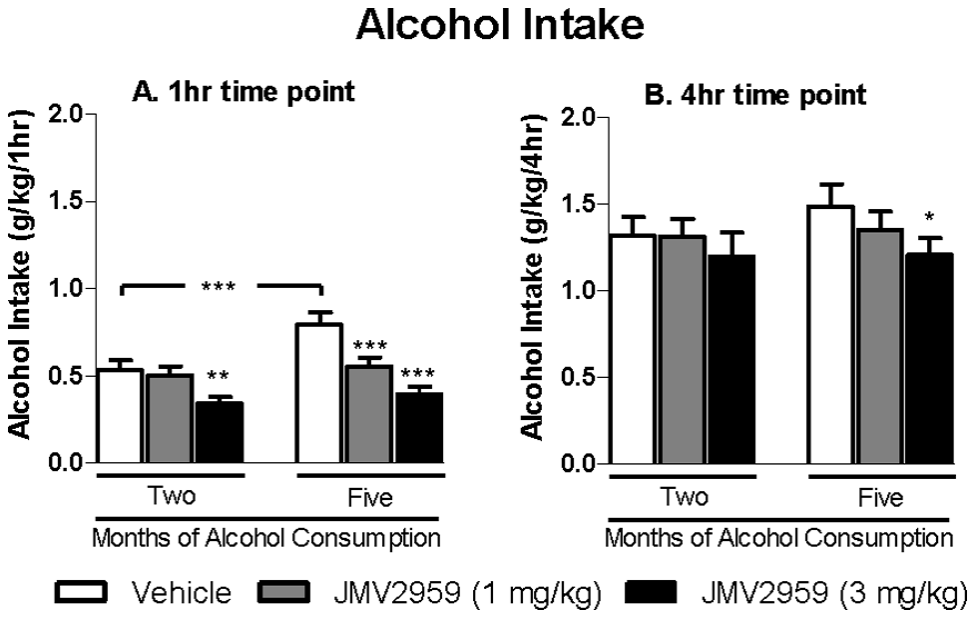
Acute JMV2959 treatment decreases alcohol intake in rats. (A) The acute effects of the GHS-R1A antagonist JMV2959 (1 or 3 mg/kg) administration on alcohol intake was evaluated at 1 hour time interval after both two and five months of voluntary alcohol consumption. (B) The acute effects of the GHS-R1A antagonist JMV2959 (1 or 3 mg/kg) administration on alcohol intake was evaluated at 4 hour time interval after both two and five months of voluntary alcohol consumption. JMV2959 significantly decreases voluntary alcohol consumption compared to vehicle and the effect was more pronounced after five compared to two months of alcohol consumption. All values represent mean alcohol intake (g/kg) ± SEM (n = 19, *P<0.05, **P<0.01, ***P<0.001 compared to corresponding vehicle or as indicated).

The acute effect of the GHS-R1A antagonist, JMV2959 on voluntary intake of alcohol was evaluated in Rcc Han Wistar rats (n = 19) after two, and subsequently, five months of voluntary alcohol consumption. JMV2959 (1 or 3 mg/kg) or vehicle was administered 20 minutes before the subjects were given access to alcohol and water. Measurements were taken after 1, 4 and 24 hours of alcohol access. Statistical analysis of the alcohol intake following acute JMV2959 administration showed an overall main effect of Treatment at both the 1 hr and 4 hr time points [1 hr: *F*(2,36) = 17.0, *P*<0.001; 4 hr: *F*(2,36) = 3.7 , *P*<0.05 ], however, there was no significant overall main effect at the 24 hr time point [F(2,36) = 0.55 , non-significant (n.s.)]. In addition, there was an overall main effect of Test Occasion at the 1 hr but not at the 4 hr nor the 24 hr time point [1 hr: *F*(1, 18) = 17.6, *P* = 0. 001; 4 hr: *F*(1, 18) = 1.4, n.s. ; 24 hr: *F*(1,18) = 4.0, n.s.]. Additionally, there was a significant interaction of Treatment*Test Occasion at the 1 hr and 4 hr but not the 24 hr time point [1 hr: *F*(2, 36) = 7.1, *P*<0.01; 4 hr: *F*(2,36) = 0.7, n.s; 24 hr: *F*(2,36) = 0.08, n.s.]. Planned comparisons further revealed that the alcohol intake was higher following vehicle treatment after five, compared to two months of alcohol consumption at the 1 hr but not the 4 hr time point (Figure 1). Additionally, after two months of alcohol consumption, the highest JMV2959 dose (3mg/kg) significantly reduced the voluntary intake of 20% alcohol compared to vehicle at the 1 hr (Figure 1A, left panel) but not the 4 hr time point (Figure 1B, left panel). After five months of alcohol consumption, both doses of JMV2959 significantly decreased the alcohol intake compared to vehicle at the 1 hr time point (Figure 1A, right panel) and the highest dose (3 mg/kg) significantly decreased the alcohol intake compared to vehicle at the 4 hr time point (Figure 1B, right panel).

**Figure 2:**
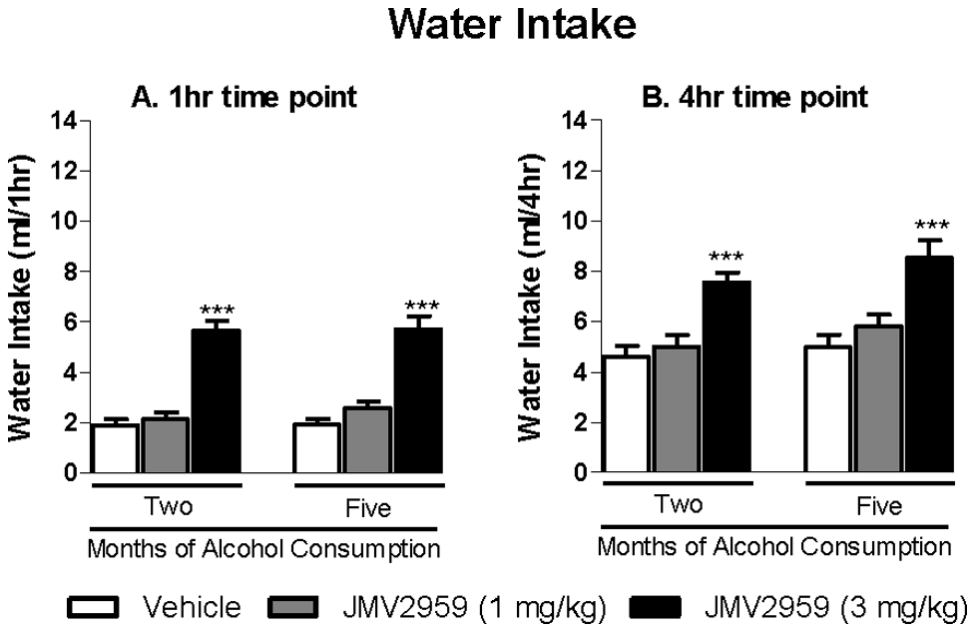
Acute JMV2959 treatment increases water intake in rats. (A) Acute administration of the GHS-R1A antagonist JMV2959 (3 mg/kg) significantly increased water consumption compared to vehicle at 1 hour time interval in rats voluntarily consuming alcohol for two and five months before the treatment. (B) Acute administration of the GHS-R1A antagonist JMV2959 (3 mg/kg) significantly increased water consumption compared to vehicle at 4 hour time interval in rats voluntarily consuming alcohol for two and five months before the treatment. All values represent mean water intake (ml) ± SEM (n = 19, ***P<0.001 compared to corresponding vehicle).

To analyse JMV2959's selectivity for alcohol, we evaluated its effect on the water intake. Statistical analysis of the water intake following JMV2959 administration showed an overall main effect of Treatment at all time points; [1 hr: *F*(2 ,36) = 75, *P*<0. 001; 4 hr: *F*(2, 36) = 38, *P*<0. 001; 24 hr: *F*(2,36) = 7.2, *P*<0. 01]. In addition, there was an overall main effect of Test Occasion at the 4 hr, but not at the 1 hr and 24 hr time points following JMV 2959 administration [1 hr: *F*(1, 18) = 0.84, n.s.; 4 hr: *F*(1,18) = 6.4, *P*<0.05 ; 24 hrs: *F*(1, 18) = 0.05, n.s.]. Furthermore, there was no significant interaction of Treatment*Test Occasion at any time point [1 hr: *F*(2,36) = 0.44, n.s.; 4 hr: *F*(2,36) = 0.34, n.s.; 24 hr: *F*(2,36) = 0.35, n.s.]. Planned comparisons revealed that the highest JMV2959 dose (3 mg/kg) significant increased the water intake compared to vehicle at the 1 hr (Figure 2A) and 4 hr (Figure 2B) time points at both test occasions (i.e. two and five months of alcohol consumption). There was no significant difference between water intake following vehicle treatment at the 4 hr time point after two compared to five months of alcohol consumption (Figure 2B).

**Figure 3:**
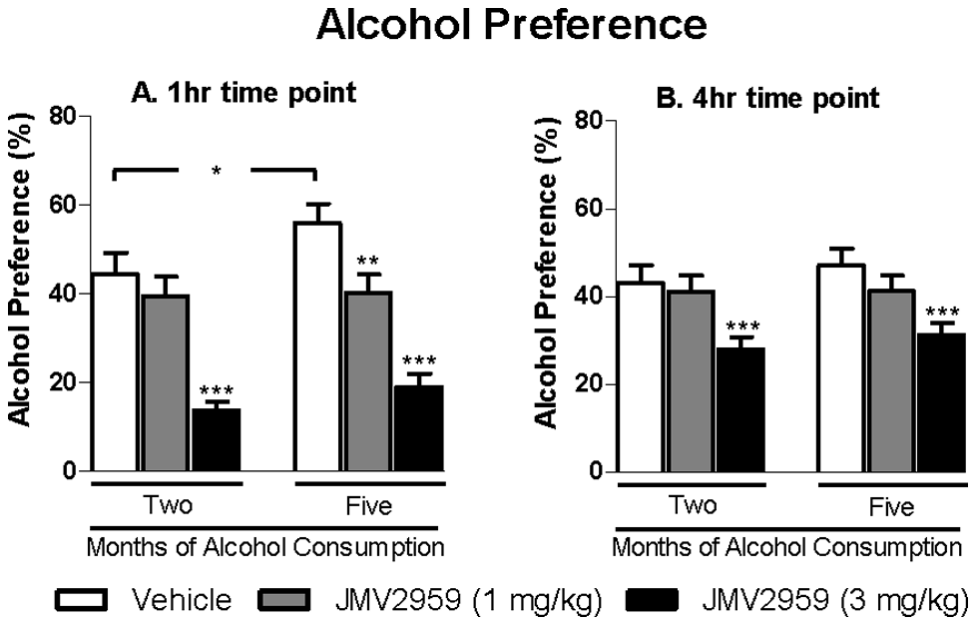
Acute JMV2959 treatment decreases alcohol preference in rats. (A) Acute administration of the GHS-R1A antagonist JMV2959 (1 or 3 mg/kg) decreases the alcohol preference compared to vehicle at 1 hour time interval in rats voluntarily consuming alcohol for two and five months before the treatment. Acute administration of the GHS-R1A antagonist JMV2959 (1 or 3 mg/kg) decreases the alcohol preference compared to vehicle at 4 hour time interval in rats voluntarily consuming alcohol for two and five months before the treatment. All values represent mean alcohol preference (%) ± SEM (n = 19, *P<0.05, **P<0.01, ***P<0.001 compared to corresponding vehicle or as indicated).

**Figure 4:**
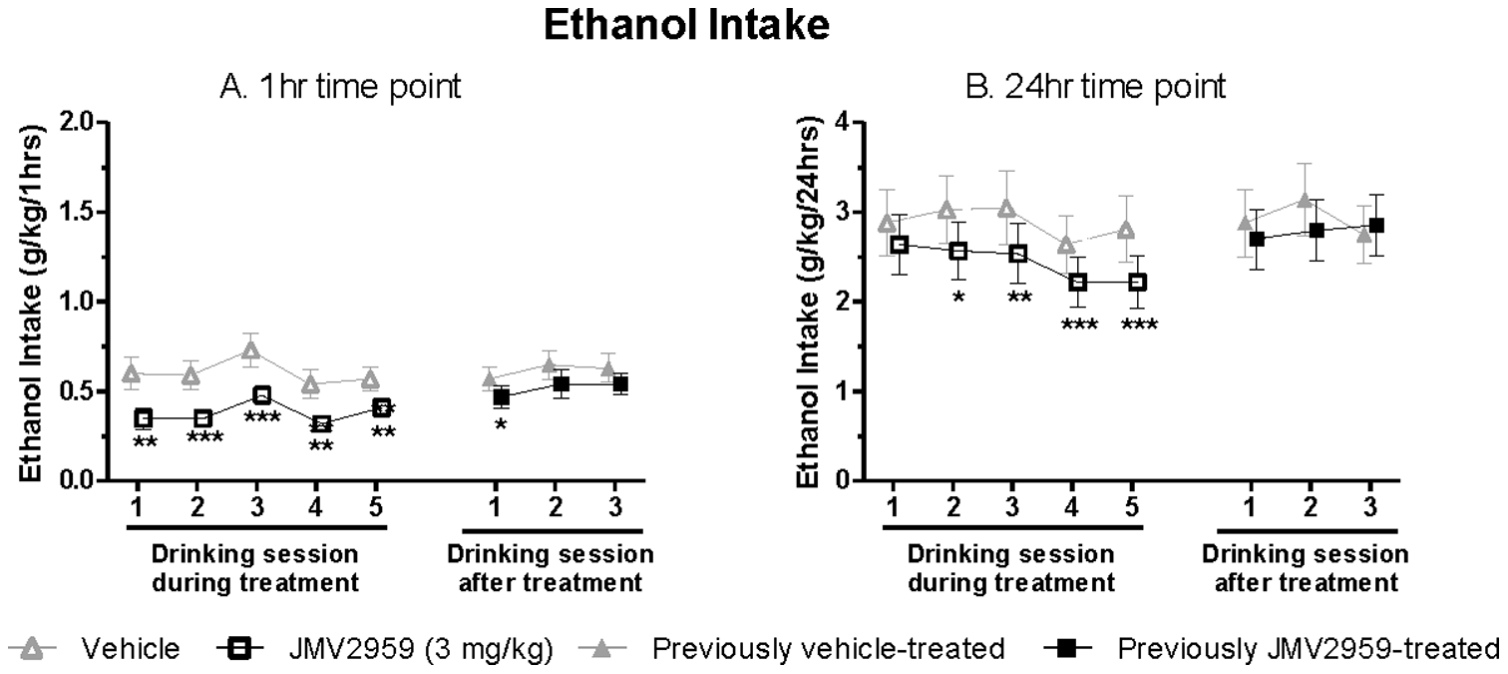
Repeated JMV2959 treatment decreases alcohol intake in rats. (A) Repeated administration of the GHS-R1A antagonist JMV2959 (3 mg/kg) significantly decreased voluntary alcohol consumption compared to vehicle at 1 hour time interval in rats voluntarily consuming alcohol for eight months before the treatment. (B) Repeated administration of the GHS-R1A antagonist JMV2959 (3 mg/kg) significantly decreased voluntary alcohol consumption compared to vehicle at 24 hour time interval in rats voluntarily consuming alcohol for eight months before the treatment. No tolerance to treatment was observed and no rebound drinking following discontinuation of treatment was observed. All values represent mean alcohol intake (g/kg) ± SEM (n = 19, *P<0.05, **P<0.01, ***P<0.001 compared to vehicle on the corresponding treatment day).

As a consequence of the significantly decreased alcohol and increased water intake following JMV2959 treatment, there was an overall main effect on Treatment when analysing the preference for alcohol over water at all time points. [1 hr: *F*(2 ,36) = 39, *P*<0.001; 4 hr: F(2,36) = 23, *P*<0. 001; 24 hr: *F*(2,36) = 5.1, *P*<0.05]. In addition, there was an overall main effect of Test Occasion at the 1 hr and 24 hr, but not the 4 hr time point [1 hr: *F*(1, 18) = 6.5, *P*<0.05; 4 hr: *F*(1,18) = 1.4 , n.s.; 24 hr: *F*(1,18) = 12, *P*<0. 01]. No significant interaction of Treatment*Test Occasion was found at any time point [1 hr: *F*(2,36) = 1.6, n.s.; 4 hr: *F*(2,36) = 0.38, n.s.; 24 hr: *F*(2,36) = 0.73, n.s.]. Planned analysis revealed that the alcohol preference was significantly increased following vehicle treatment after five compared to two months of alcohol consumption (Figure 3A). Furthermore, the highest JMV2959 dose (3 mg/kg) significantly decreased the alcohol preference compared to vehicle at all time points after both two and five months of alcohol consumption (1 hr (Figure 3A) and 4 hr (Figure 3B); 24 hr: data not shown). In addition, after five months of alcohol consumption, also the lowest JMV2959 dose (1 mg/kg) significantly decreased the preference compared to vehicle (Figure 3A, right panel).

### Repeated JMV2959 treatment selectively decreases alcohol intake without inducing tolerance during treatment or rebound increase in alcohol intake after discontinuing the treatment

A key aspect to determine the potential of a novel treatment for alcohol dependence is whether repeated treatment will remain effective. The repeated JMV2959 (3 mg/kg) treatment in rats that had voluntarily consumed alcohol for approximately eight months, induced an overall main effect of Treatment [1 hr: *F*(1,18) = 31, *P*<0.001; 4 hr: *F*(1,18) = 17, *P* = 0.001; 24 hr: *F*(1,18) = 13, *P*<0.01] and Day [1 hr: *F*(7,126) = 5.1, *P*<0.001; 4 hr: *F*(7,126) = 4.9, *P*<0.001; 24 hr: *F*(7,126) = 5.3, *P*<0.001] at all time points. In addition, there was a significant interaction of Treatment*Day at the 1 hr and 24 hr time-point [1 hr: *F*(7,126) = 2.3, *P*<0.05; 4 hr: *F*(7,126) = 1.2, n.s; 24 hr: *F*(7,126) = 2.2, *P*<0.05]. Thus, planned comparison was conducted at the 1 hr and 24 hr time point. JMV2959 significantly decreased alcohol intake compared to vehicle on each of the five alcohol-treatment days at the 1 hr time point (Figure 4A). At the 24 hr time point, there was a significant decrease in alcohol intake compared to vehicle on all the treatment days except the first (Figure 4B). The alcohol intake remained significantly lower compared to vehicle during the first drinking session post treatment at the 1 hr time-point (Figure 4A). At the following two drinking sessions at the 1 hr time point and at all three sessions post treatment at the 24 hr time point, there was no difference in alcohol intake between JMV2959 and vehicle pre-treatment (Figure 4).

**Figure 5:**
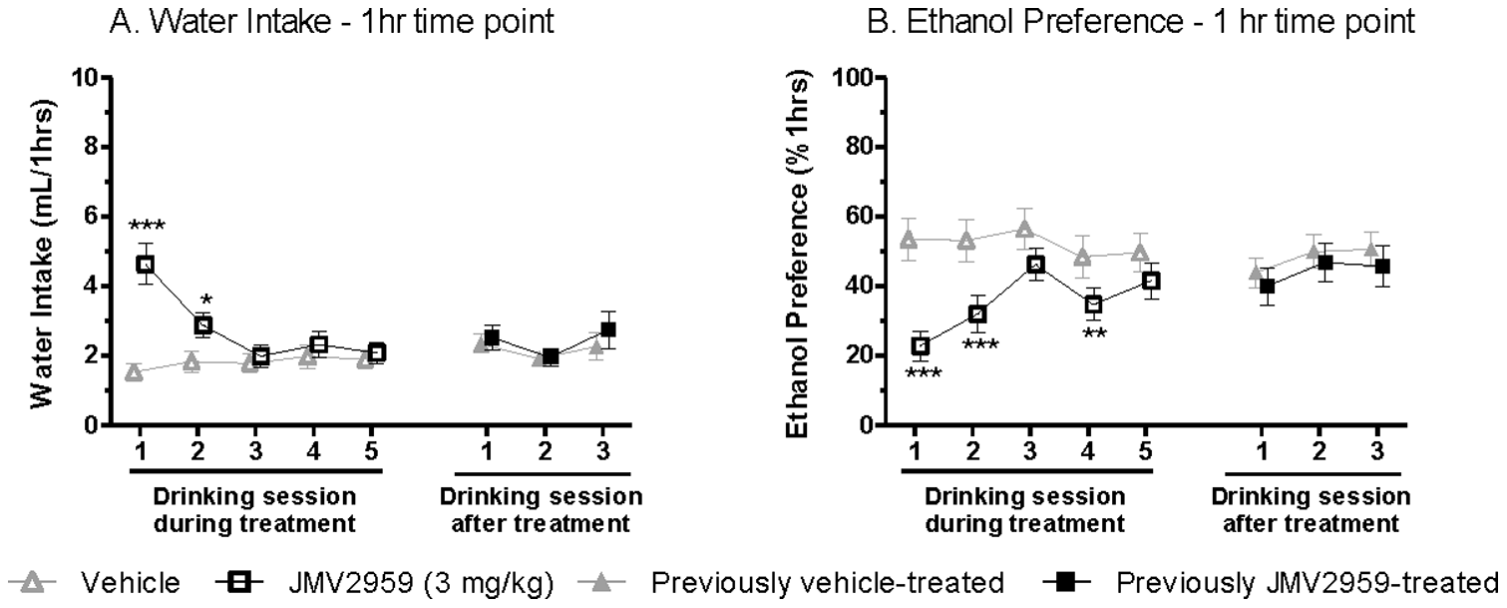
Repeated JMV2959 treatment increases water intake in rats. (A) Repeated administration of the GHS-R1A antagonist JMV2959 (3 mg/kg) significantly increased water intake compared to vehicle at the 1 hour time interval in rats voluntarily consuming alcohol for eight months before the treatment. (B) Repeated administration of the GHS-R1A antagonist JMV2959 (3 mg/kg) significantly decreased alcohol preference compared to vehicle at the 1 hour time interval in rats voluntarily consuming alcohol for eight months before the treatment. All values represent mean ± SEM (n = 19, *P<0.05, **P<0.01, ***P<0.001 compared to vehicle on the corresponding treatment day).

When analysing the water intake on the days with alcohol access, the repeated JMV2959 (3 mg/kg) treatment induced an overall main effect of Day [1 hr: *F*(7,126) = 4.0, *P* = 0.001; 4 hr: *F*(7,126) = 16, *P*<0.001; 24 hr: *F*(7,126) = 4.3, *P*<0.001] at all time points, but of Treatment only at the 1 hr time point [1 hr: *F*(1,18) = 16, *P* = 0.001; 4 hr: *F*(1,18) = 4.2, n.s.; 24 hr: *F*(1,18) = 1.8, n.s.]. In addition, there was a significant interaction of Treatment*Day at the 1 hr and 4 hr time-point [1 hr: *F*(7,126) = 6.5, *P*<0.001; 4 hr: *F*(7,126) = 3.9, *P* = 0.001; 24 hr: *F*(7,126) = 0.47, n.s]. Planned comparison at the 1 hr time point further revealed that the water intake was significantly increased compared to vehicle only at the first two treatment days (Figure 5A).

Analysis of alcohol preference revealed a significant overall main effect of Treatment [1 hr: *F*(1,18) = 3.3, *P*<0.01; 4 hr: *F*(1,18) = 25, *P*<0.001.; 24 hr: *F*(1,18) = 12, *P*<0.01] and Day [1 hr: *F*(7,126) = 4.0, *P* = 0.001; 4 hr: *F*(7,126) = 3.8, *P* = 0.001; 24 hr: *F*(7,126) = 2.3, *P*<0.05] at all time points. However, there was a significant interaction of Treatment*Day only at the 1 hr and 4 hr time point [1 hr: *F*(7,126) = 4.7, *P*<0.001; 4 hr: *F*(7,126) = 3.2, *P*<0.01; 24 hr: *F*(7,126) = 1.6, n.s]. Thus, planned comparison was conducted only at the 1 hr and 4 hr time points. Alcohol preference was significantly decreased compared to vehicle on treatment day 1, 2 and 4 at the 1 hr and treatment day 1, 4 and 5 at the 4 hr time point, respectively (1 hr: Figure 5B; 4 hr: data not shown). During each of the five drinking sessions post JMV2959 treatment, there was no significant difference in preference compared to vehicle (1 hr: Figure 5B; 4 hr: data not shown).

### Effects of acute treatment with the GHS-R1A antagonist JMV2959 on alcohol intake using the alcohol deprivation effect in rats

The effect of acute JMV2959 treatment on relapse drinking in the alcohol deprivation paradigm was investigated in a separate group of rats (n = 15 per treatment group). After seven weeks of intermittent alcohol intake, there was no significant difference in 24 hour baseline consumption of alcohol (vehicle 2.57±0.32 g/kg; JMV2959 2.68±0.29 g/kg; [*F*(1,28)  = 0.07, n.s.]), food (vehicle 22.36±0.59 g; JMV2959 22.69±0.71 g; [*F*(1,28)  = 0.12, n.s.]) or total fluid (vehicle 26.25±0.77 g; JMV2959 25.11±0.59 g; [*F*(1,28)  = 1.37, n.s.]) between the two groups of rats that would be subjected to JMV2959 or vehicle treatment in the alcohol deprivation experiment.

**Figure 6:**
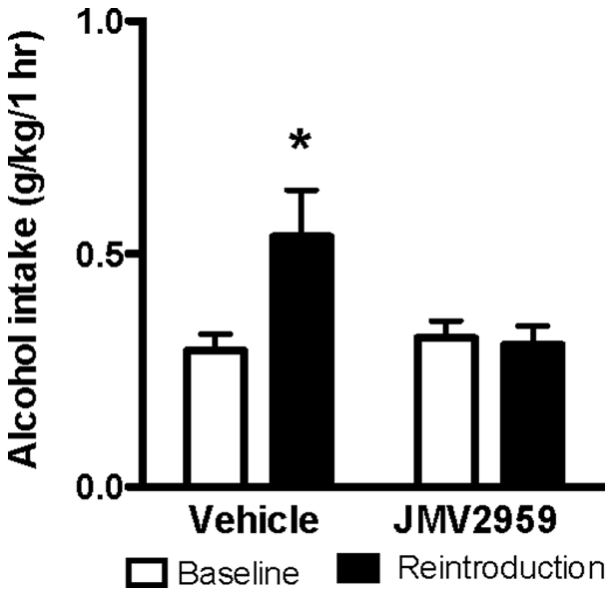
Acute JMV2959 treatment prevents the alcohol deprivation effect in rats. Following seven weeks of alcohol consumption (baseline, white bars) the rats were exposed to ten days of alcohol abstinence. Before alcohol was reintroduced (black bars) the rats were treated with wither JMV2959 or vehicle. An alcohol deprivation effect was observed in vehicle treated rats but not in rats treated with the GHS-R1A antagonist, JMV2959. Data are presented as mean alcohol intake (g/kg/1 hr) ± SEM. (n = 15 in each group, *P<0.05 compared to corresponding baseline).

After ten days of forced alcohol abstinence, the rats were treated with JMV2959 (n = 15) or vehicle (n = 15) 20 minutes before given access to one bottle of water and one bottle of 20% alcohol. After 1 hour of alcohol access there was an overall main effect of treatment [*F*(1,28) = 7.44, *P* = 0.011 ] and an significant interaction of treatment x time [*F*(1,28) = 9.54, *P* = 0.005]. However, no effect of time was observed [*F*(1,28) = 1.98, n.s.]. Posthoc analysis revealed a significant alcohol deprivation effect (*i.e.* significant increase in alcohol intake compared to respective baseline) in vehicle (*P*<0.05) but not JMV2959 (n.s.) treated rats (Figure 6). In addition, the JMV2959 treatment significantly reduced alcohol intake (g/kg) (vehicle 0.5±0.2; JMV2959 0.3±0.2; *P*<0.05 and alcohol preference (vehicle 26±5; JMV2959 13±2; *P*<0.05) as well as significantly increased water intake (ml) (vehicle 4.4±0.5; JMV2959 5.7±0.5; *P* = 0.05) during the first hour compared to vehicle treatment. However, there were no significant differences in total fluid intake (vehicle 5.8±0.4; JMV2959 6.1±0.5; n.s.), food intake (vehicle 2.9±0.5; JMV2959 1.9±0.4; n.s.) or body weight (Vehicle: 428±7 g and JMV2959: 426±9, n.s.) following the different treatments.

After 24 hrs of alcohol access, there were no overall main effects of treatment on alcohol intake (g/kg) (vehicle 13.0±0.4; JMV2959 3.2±0.3; n.s.), water intake (ml) (vehicle 21.1±1.5; JMV2959 18.8±0.7; n.s.), alcohol preference (%) (vehicle 28±4; JMV2959 31±3; n.s.), food intake (g) (vehicle 22.0±0.9; JMV2959 20.2±0.8; n.s.) or total fluid intake (ml) (vehicle 29.2±1.0; JMV2959 27.2±0.8; n.s.).

During the three drinking days following the alcohol deprivation experiment (during which the rats were untreated), no difference was observed at the 24 hrs time point in alcohol intake (g/kg) (vehicle 1.9±0.2; JMV2959 2.1±0.2; *F*(1,28) = 0.52 , n.s.), food intake (g) (vehicle 20.5±0.6 g; JMV2959 19.7±0.6; *F*(1,28) = 096, n.s.), water intake (ml) (vehicle 19.9±1.6 g; JMV2959 18.2±1.7; *F*(1,28) = 0.54, n.s.) or total fluid intake (ml) (vehicle 24.4 g±0.9 JMV2959 22.4±1.0) [*F*(1,28)  = 2.61, n.s.) between the previous treatment groups.

### Gene expression in rats voluntarily consuming alcohol for ten months

**Figure 7:**
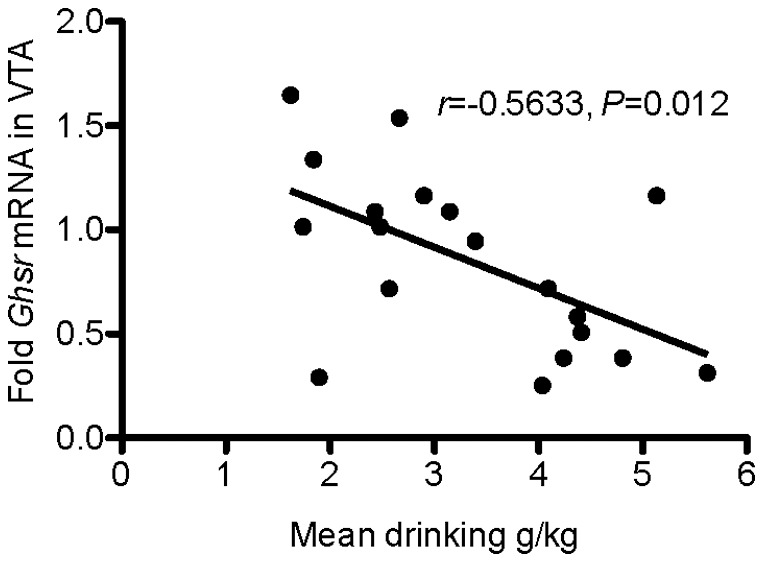
Scatter plot of Ghsr expression in the VTA. The present figure shows a significant negative correlation between the expression of *Ghsr* in the VTA (data presented as arbitrary units 2^−ΔΔC^_T_ where the mean ΔC_T_ of the group of low alcohol consuming rats was set as the calibrator) and mean alcohol consumption (g/kg/24 hrs) in rats that have voluntarily been drinking alcohol for ten months.

To evaluate the effects of long-term alcohol consumption on *Ghsr* expression, the 19 rats that voluntarily had consumed alcohol for ten months and previously had undergone acute, and subsequently repeated JMV2959 treatment were divided into low- and high-consumers based on their level of alcohol consumption (low consumers: 2.3±0.2 g/kg/24 hr; high consumers: 4.5±0.2 g/kg/24 hr). In the VTA, there was a significant effect of alcohol consumption with higher *Ghsr* expression in the low- compared to the high-consuming rats (Low consumers: 1.10±0.39; High consumers: 0.58±0.31; *t*(17) = 3.12, *P* = 0.006, *P_c_* = 0.031; Table 1). Further analysis of the data revealed a significant negative correlation between *Ghsr* expression and alcohol intake (g/kg/24 hrs) (Pearson Correlation *r* = −0.56; n = 19; *P* = 0.012; *P*_c_ = 0.047; Figure 7). No significant effect was found on *Ghsr* gene expression in the N.Acc., PFC, hippocampus or amygdala (Table 1), or on the expression of the α3, α4 and β2 nicotinic acetylcholine receptor subtype genes in any of the brain regions examined (VTA, PFC, hippocampus, amygdala and N.Acc.; data not shown).

**Table 1:** *Ghsr* mRNA expression in reward related areas of low (2.3±0.2 g/kg/24 hr) and high (4.5±0.2 g/kg/24 hr alcohol-consuming rats after approximately ten months of voluntary alcohol consumption.

Brain region	Low alcohol consuming rats	High alcohol consuming rats	t	df	Effect^1^	*P*-value^2^	*P*_c_-value^3^
	n = 10	n = 9					
Ventral tegmental area	1.09±0.39	0.58±0.31	3.119	17	−1.93	0.006	0.031
Nucleus Accumbens	1.93±2.57	2.19±2.67	−0.212	17	1.50	0.83	1
Amygdala	1.75±2.83	2.78±2.27	−0.872	17	2.15	0.40	1
Hippocampus	1.34±1.35	1.33±1.10	0.024	17	1.06	0.98	1
Prefrontal cortex	3.80±8.85	3.50±3.50	0.097	17	2.15	0.92	1

Data presented as mean arbitrary units 2^−ΔΔC^_T_ ± SD, where the mean ΔC_T_ of the group of low alcohol consuming rats was set as the calibrator.

1 estimated effects of high alcohol consumption on *Ghsr* expression presented as up or down folds.

2 significant levels obtained by independent samples T-test.

3 significant levels adjusted for multiple comparisons.

### Methylation analysis in high- and low-alcohol consuming rats

The effects of long-term alcohol consumption on *Ghsr* methylation in the VTA in the rats (n = 19) that voluntarily had consumed alcohol for ten months and previously had undergone acute, and subsequently repeated JMV2959 treatment was investigated. The methylation assay for *Ghsr* in the VTA was successfully run in eight of the ten low-alcohol consuming rats and in all of the high-alcohol consuming rats. No significant difference in methylation degree was observed neither for any of the individual CpG sites nor for overall methylation between the high and low consuming rats after ten months of voluntary alcohol consumption (Low consumers: 5.42±0.91%; High consumers: 5.52±0.78%; *t*(15) =  −0.241, *P* = 0.81; Table 2). Furthermore, no correlation was found between the overall methylation degree and gene expression of the *Ghsr* in the VTA (*Pearson Correlation r* = −0.11, *P* = 0.67).

**Table 2:** Data expressed as methylation degree (%±SD), both overall for the CpG island and at the individual CpG sites, of the *Ghsr* in ventral tegmental area of low (2.3±0.2 g/kg/24 hr) and high (4.5±0.2 g/kg/24 hr) alcohol-consuming rats after approximately ten months of voluntary alcohol consumption.

	Low alcohol consuming rats	High alcohol consuming rats	*P*-value^1^	*P*_c_-value^2^	t	df
	n = 8	n = 9				
**Overall methylation**	5.42±0.91	5.52±0.78	0.81	1	−0.24	15
**CpG 1**	7.92±0.96	8.05±1.61	0.85	1	−0.19	15
**CpG 2**	4.89±0.91	5.57±0.84	0.13	0.52	−1.60	15
**CpG 3**	5.17±1.11	5.08±0.63	0.84	1	0.21	15
**CpG 4**	3.70±1.06	3.38±1.43	0.61	1	0.52	15

1 significant levels obtained by independent samples T-test.

2 significant levels adjusted for multiple comparisons.

## Discussion

The present study provides further evidence that the GHS-R1A have an important role in regulating alcohol drinking in rats. Acute as well as repeated treatment with the GHS-R1A antagonist JMV2959 decreased alcohol intake and prevented the alcohol deprivation effect in rats. A significant down-regulation of the *Ghsr* expression in the VTA was observed in high alcohol-consuming rats compared to low alcohol-consuming rats after approximately ten months of voluntary alcohol consumption. However, no differences in methylation degree were found between these rats nor did we find a correlation between overall methylation degree and *Ghsr* expression in the VTA.

While previous studies have shown that ghrelin and its receptor are physiological regulators of food intake and body weight homeostasis [11]–[13], [46], the present study gives support to the hypothesis that GHS-R1A antagonists has potential as novel treatments for alcohol dependence. Firstly, the ability of the GHS-R1A antagonist JMV2959 to decrease voluntary alcohol intake was more pronounced following long-term alcohol consumption. Indeed, we showed that both JMV2959-doses decreased the alcohol intake compared to vehicle after five months of alcohol consumption, whereas only the highest dose (3 mg/kg) was effective after two months. In addition, the effect of JMV2959 was more long lasting after eight (effect up to 24 hours) when compared to both five and two months (effect up to 4 and 1 hour, respectively) of alcohol consumption. Secondly, repeated JMV2959 treatment reduces alcohol intake significantly compared to vehicle each treatment day, indicating that no tolerance to JMV2959's ability to reduce alcohol consumption develops. Thirdly, we showed that the GHS-R1A antagonist did not induce a rebound increase in alcohol intake after the treatment was terminated. Finally, we show to our knowledge for the first time that acute JMV2959 treatment in rats prevents the important characteristics of alcohol dependence, namely the alcohol deprivation effect. The alcohol deprivation effect in rodents has been suggested to reflect relapse caused by craving in the clinical setting [38]. Indeed, the currently available agents for treatment of alcohol dependence, naltrexone and acamprosate, prevents both the alcohol deprivation effect in rats [47], [48] and craving-induced relapse in humans [49]. Given that high plasma ghrelin levels are associated with craving in patients with alcohol dependence [50]–[52] a tentative explanation of the present finding that JMV2959 attenuates the alcohol deprivation effect may be that JMV2959 blocks the ability of ghrelin to activate the mesolimbic dopamine system and induce craving. This hypothesis finds support in a recent longitudinal clinical study that showed not only that base-line ghrelin levels were highly positively correlated with self-reported craving scores in alcohol dependent individuals but also that blood ghrelin levels increased in alcohol dependent subjects who abstained from alcohol when compared to non-abstinent alcohol dependent subjects [51]. Collectively the results from the present study support previous studies indicating a role for ghrelin signalling in alcohol-mediated behaviours. For example, we have previously shown that suppression of the ghrelin signalling system attenuates the rewarding properties of alcohol as measured by locomotor stimulation, accumbal dopamine release and condition place preference in mice [7], [8]. Moreover, GHS-R1A antagonists (administered systemically or centrally) reduce, whereas ghrelin increase alcohol intake as well as operant self-administration of alcohol in rodents [7], [9], [10]. Additionally, one single nucleotide polymorphism in the GHS-R1A gene has been associated with high alcohol consumption in humans [53] and there are associations between haplotypes of the preproghrelin and GHS-R1A genes and paternal alcohol dependence as well as type II alcohol dependence in a Swedish female alcohol dependent population [54]. In addition to alcohol mediated behaviours, the rewarding properties of addictive drugs as well as palatable food are mediated via ghrelin signalling in rodents [23]–[29], [41], [55], [56].

In the present study, GHS-R1A expression was detected in VTA, N.Acc. amygdala, hippocampus and PFC in accordance with previous studies [15], [16], [57]. However, to our knowledge the present study is the first investigating the *Ghsr* expression following ten months of voluntary alcohol consumption. The expression of *Ghsr* was significantly lower in the VTA of rats voluntarily consuming high compared to low amounts of alcohol. Further analysis revealed a negative correlation between *Ghsr* expression in the VTA and alcohol consumption. The finding that there was no significant difference in *Ghsr* expression in any other brain regions examined together with our previous findings that local administration of ghrelin into the VTA increases alcohol intake in mice [7], supports our hypothesis that within the VTA GHS-R1A are central for modulating alcohol intake. This hypothesis is further supported by previous studies showing that local administration of GHS-R1A antagonists into the VTA attenuates ghrelin-induced reward [58] as well as ghrelin-mediated sucrose intake in rodents [59]. The ability of GHS-R1A to heterodimerize with dopamine D1 and D2 receptors in the VTA [60], [61] as well as the constitutive activity of the GHS-R1A [62] have been suggested to alter the sensitivity of the mesolimbic dopamine system. We therefore hypothesize that within the VTA, GHS-R1A regulates the ability of alcohol and other addictive drugs to activate the mesolimbic dopamine system. Moreover, the findings in the present study showing a negative correlation between *Ghsr* expression in the VTA and alcohol consumption indicate that the long-term alcohol consumption may change the sensitivity of ghrelin systems within the VTA. This may be a tentative explanation to why the efficacy of JMV2959 to decrease voluntary alcohol intake is more pronounced following long-term alcohol consumption.

In the present study there was no difference in the methylation degree in the studied CpG island of the *Ghsr* between rats voluntarily consuming high or low amounts of alcohol for approximately ten months. Moreover, no correlation between methylation degree and *Ghsr* expression in the VTA was found. Previous studies have shown that methylation can change expression patterns [63] and that such epigenetic mechanisms could explain the complexity of psychiatric complex disorders including alcohol dependence [64]. However, the observed down-regulation of the *Ghsr* gene following voluntary consumption of high amounts of alcohol appears to be independent of epigenetic control.

Ghrelin and alcohol partly share common neurochemical mechanisms, since α3β2* rather than α4* subtypes of the nicotinic acetylcholine receptor mediate both ghrelin and alcohol reinforcement [34]–[36]. Both preclinical and clinical findings indicate that α3β2*subtype is a potential target for treatment of alcohol dependence. Indeed, the stimulatory, rewarding, dopamine-enhancing and anticipatory properties of alcohol as well as alcohol intake has previously been shown to involve central nicotinic acetylcholine receptor, especially the α3β2* rather than α4* subtypes located in the VTA [35], [36], [65]–[68]. Moreover, pharmacological suppression of the α3β2 subtypes, e.g. by using the smoking secession medication varenicline, reduced alcohol intake in rodents [35], [37] and in heavy-drinking smokers in a human laboratory [69] as well as heavy-drinking smokers and alcohol dependent individuals in double-blinded placebo studies [70], [71]. However, in the present study, no associations between the expression of the α3, α4 and β2 subtypes and alcohol intake were found in any of the examined reward related areas. One possible explanation for the discrepancy between the present results and previous studies could be that the rats in the present study had voluntarily consumed alcohol for approximately ten months while the rodent in previous studies either were alcohol naïve or exposed to alcohol for short periods of time.

The present study provides further evidence for a connection between the central ghrelin signalling system, possibly at the level of VTA, and alcohol consumption as well as relapse. Collectively, the GHS-R1A appear to be important for alcohol mediated behaviours in rodents and humans and these receptors are therefore promising new candidate targets for pharmacological treatment of alcohol dependence in humans.
